# Pain Narratives in Breast Cancer Survivors

**DOI:** 10.1155/2012/153060

**Published:** 2012-10-24

**Authors:** Patrick Peretti-Watel, Marc-Karim Bendiane, Laura Spica, Dominique Rey

**Affiliations:** ^1^INSERM, UMR912 (SESSTIM), 13006 Marseille, France; ^2^Aix Marseille Université, UMR_S912, IRD, 13006 Marseille, France; ^3^ORS PACA, Observatoire Régional de la Santé Provence-Alpes-Côte d'Azur, 13006 Marseille, France

## Abstract

In-depth interviews were conducted with French breast cancer survivors 24 month after cancer diagnosis (*N* = 21 women). We documented their experience of chronic pain, compared their pain narratives with their answers to the WHOQOL-BREF questionnaire, and studied both the meaning they gave to their pain and how they dealt with it in their daily lives. Half of participants reported are suffering from iatrogenic chronic pain. Most of the time, this pain was not captured by the WHOQOL questionnaire and was not medically treated. Patients “normalized” their pain in various ways: they considered it either as a necessary step on the road to recovery, as the proof of treatment efficacy, or as a permanent condition one must learn to live with. They learned to deal with pain by taking precautions, giving up certain activities, and changing the way they performed others. Participants were also prone to compare themselves with other patients suffering worse pain. Breast cancer survivors should be better informed about chronic pain and how to alleviate it. Physicians should contribute to fighting pain-related beliefs which lead patients to conceal their pain. Techniques used by patients to cope with chronic pain in their daily lives should also be promoted.

## 1. Introduction

With about one million new cases in the world each year, breast cancer is the most common malignancy in women and comprises 18% of all female cancers [1]. In France, it accounts for 36% of all female cancers and its incidence is increasing sharply (+60% during the past 20 years), particularly among women aged 50–64 and probably because of improved breast cancer screening in this age group [2]. Moreover, progress in terms of screening, treatment, and care has contributed to improving the relative survival rate for this cancer (the 5 year survival rate after diagnosis is 82% in France) [3, 4]. In other words, in France as in other developed countries, an increasing number of women develop breast cancer, and among them an increasing proportion survive their cancer [5]. As a consequence, it is crucial to develop patient-based outcome measures for these women, such as quality of life (QoL). To our knowledge, most studies to date examining QoL in cancer survivors have relied on psychometric instruments, and especially tools measuring general health-related QoL, such as the WHOQOL or SF36 [6–8].

Experiencing chronic pain provokes distress and fatigue; it also impairs appetite, sleep, mood, and the ability to perform many daily living tasks [9, 10]. Thus, chronic pain is a major determinant of QoL, especially among breast cancer patients [11–13], and psychometric scales measuring QoL routinely comprise pain assessment items. Pain is also one of the most common and most feared symptoms of cancer. Cancer pain may occur at any stage of the disease, generally depending on the type of tumor, the presence, and location of metastases and less frequently on cancer treatment [14, 15]. However, in the case of breast cancer, pain is almost always iatrogenic, due to postoperative complications, radiotherapy, or chemotherapy [16]. This specificity could be of some importance regarding breast cancer survivors' understanding of and attitudes toward the chronic pain many of them endure. For example, breast cancer survivors' could be more likely to consider pain as a normal aspect of the recovery process, and giving such a meaning to their pain may affect the way they report it to either health professionals or professional interviewers.

The present paper used in-depth interviews conducted with breast cancer survivors participating in a cohort study. Its aims were two-fold. First, we documented patients' experience of chronic pain and how it affected their everyday lives. To do so, we compared in-depth interviews with results from the WHOQOL-BREF questionnaire regarding chronic pain and its impact on everyday activities. Second, in order to better understand the discrepancies revealed by this comparison, we focused on respondents' attitudes toward pain, especially in terms of how they gave meaning to it and how they dealt with it in their daily lives.

## 2. Materials and Methods

### 2.1. Participants

Participants were recruited among patients already enrolled in the cohort study ELLIPSE whose purpose was to investigate how breast cancer patients deal with posttreatment daily life. This cohort study focused on younger (aged 18–40) and older (aged 65 and over) women (*N* = 1, 200; patients recruited in southeastern France). All cohort respondents completed the WHOQOL-BREF questionnaire 24 months after diagnosis.

The World Health Organization Quality of Life (WHOQOL) questionnaire is an international cross-culturally comparable quality of life assessment instrument [17, 18]. It assesses the individual's perceptions in the context of their culture and value systems, and their personal goals, standards and concerns. This instrument has been widely field-tested. The WHOQOL-BREF is a shorter version of the original questionnaire. It comprises 26 items measuring physical health, psychological health, social relationships, and environment. We used it to compute summary scores for physical health (PHY) and psychological health (PSY) (for both scales, a higher score is an indication of better health). Questions related to physical health deal with activities of daily living, dependence on medication or medical aid, energy and fatigue, mobility, pain and discomfort, sleep, and rest, as well as work capacity. Questions related to psychological health deal with bodily image and appearance, negative/positive feelings, self-esteem, spirituality, thinking, learning, memory, and concentration.

Participants were not randomly selected. Instead, we used available data related to women already enrolled in the ELLIPSE cohort to select 21 women with contrasting ages (13 were aged between 26 and 43 and 8 were aged between 66 and 83) and contrasting PHY and PSY scores: 8 had scores significantly below the group average, 10 had scores well above the average, and 7 had average scores. A letter of information was sent to selected women asking them whether they would consider participating. A few days later, women were contacted by phone to introduce ourselves and to give further information about the study.

### 2.2. Data Collection

All contacted women agreed to participate. In-depth semistructured interviews were conducted in participants' homes. A short interview guide helped direct the conversation and interaction with the participants towards the discovery of the meaning they gave to their pain. The interview guide included the following themes: disease and treatment history, current health, experience of pain during the treatment phase, and in current daily life, relationship with health professionals. Interviews lasted between 1 and 4 hours. Interviews were tape-recorded with the patient's consent, transcribed verbatim, and observation notes were added. Any information which would indentify a participant was removed to preserve anonymity and confidentiality (first names have been changed in quotations, infra). We used an inductive approach based on grounded theory [19, 20].

### 2.3. Data Analysis

Data were analyzed concurrently with data collection: the themes emerging from the first interviews helped to refine the interview guide used for the next set of interviews, these latter interviews in turn informing the next set and so on. The study's authors coded the transcripts independently and met to compare and discuss their codes. Finally, we undertook a second round of coding to condense our set of initial thematic codes into more abstract, second-line codes.

Regarding comparison with the WHOQOL-BREF questionnaire, we focused on the following items: QoL patient self-rating (how would you rate your quality of life? Very poor, poor, neither poor nor good, good, very good); activity limitation due to pain (how much do you feel that pain prevents you from doing what you need to do? Not at all, a little, a moderate amount, very much, a great deal); satisfaction in everyday activities (how satisfied are you with your ability to perform daily living activities? Very dissatisfied, dissatisfied, neither satisfied nor dissatisfied, satisfied, very satisfied).

## 3. Results

### 3.1. Description of Participants

Table 1 provides some basic sociodemographic and medical characteristics of the 21 breast cancer survivors interviewed. Average PHY scores were higher among participants aged 26–43, although average PSY scores were similar for both younger and older women. Most women aged 26–43 were married (versus half of those aged 66–83) and working at the time of the survey (all the older women were retired). Concerning previous and current medical treatment, all participants had received chemotherapy (and only some of the younger women received taxane chemotherapy). All participants also received surgery: 15 had a partial mastectomy (lumpectomy), 6 had a total mastectomy, and 13 had axillary surgery. Breast reconstruction was more frequent among younger participants (9 out of 13 received it, versus 4 out of 8 among the older ones). At the time of the study, almost all women (18 out of 21) were on hormonal therapy (usually, aromatase inhibitors are prescribed to old patients, while the younger ones receive tamoxifen).

### 3.2. Chronic Pain in the WHOQOL-BREF and In-Depth Interviews

Ten of the 21 participants reported daily chronic pain at the time of the survey (see Table 2). Reported chronic pain was breast cancer treatment-related, mainly due to postoperative complications (scar pain, arm lymphoedema) and hormonotherapy side-effects (muscular and bone pain). In one case (Nancy), the pain resulted from the combination of treatment side-effects (when a tight scar became painful due to weight gain induced by hormonal therapy). Among these 10 participants reporting pain, only 5 were taking painkillers at the time of the interview (paracetamol, diclofenac—which is a nonsteroidal anti-inflammatory drug, and in one case trinitrin for chest pain). Pain hindered several participants from performing ordinary daily activities, such as lifting a pack of milk, holding a handbag, or going upstairs.

Despite their daily chronic pain, only 3 of the 10 women stated that their quality of life was “poor” or “very poor”. Only two felt “very much” that pain prevented them from doing what they need to do and only one was “not satisfied at all” with her ability to perform daily living activities. The case of Emily illustrated the typical discrepancy between responses to the WHOQOL-BREF items and discussions about one's pain during in-depth interviews. She rated her QoL as “very good”, she felt only “a little” activity limitation due to pain, and she stated being satisfied with her ability to perform daily living activities. But during the in-depth interview, she reported always being out of breath since the completion of chemotherapy, and also being unable to ride a scooter, to raise her arms, or even to lift a pack of milk, because of her arm pain.

### 3.3. The Various Meanings of Pain

Participants reported contrasting experiences and attitudes toward pain, depending on how they perceived it. For some women who reported chronic arm and muscular pain, bearing this pain was considered as a necessary step in fighting their disease experience. They viewed pain as an ordeal they must go through to get cured. This meaning justified the absence of painkillers:

*It was a battle between me and the pain. I wanted to win without cheating. It is an on-going fight, make no mistake! That's why there are people who escape and others who cannot.*  (Nancy) **

Conversely, other women reported that they did not have to suffer since they already felt cured of their cancer:

*I consider that I am healed; so I do not need to feel pain… *(…)*…To be down-to-earth about it, I am sick, I am operated on, I am healed and it's over. And if I am cured I do not really have any reason to feel pain.*  (Hillary) **

Participants' attitudes toward pain and pain management also depended on whether they believed pain was transitory and destined to disappear, or whether they considered it as a new and permanent condition. In the latter case, it was found that some women refused painkillers because they did not want to take them for the rest of their lives:

** [Do you feel pain all the time?]  *Oh yes, it's a pain that doesn't go away! We must learn to live with it. Then it becomes almost normal. I feel pain, it's that simple. That doesn't change.*  (Emmy) **

** [Is the pain going to disappear?]  *No, I don't know, I don't think so. I was operated on two years ago. If it was going to disappear, I think it would already be gone, the pain.*  [And your doctor, what did he say to you about your pain?]  *He prescribed me physical therapy. Anyway, I won't take drugs.*  [But he proposed them to you?]  *Yes, certainly, I don't know. He probably gave me a prescription but I didn't get the drugs. I don't need medication. I must learn to live with pain. Because I don't want to take drugs forever.*  (Bree) **

**[Do you take drugs for relief?]  *No. I've had enough of drugs. No, I don't take anything. Nothing at all.*  [Not even to relieve the pain in your arm?]  *No, do-not-want-anything-at-all. I do not want drugs.*  [You've taken too much?]  *Yes. Yes. But I mean… otherwise, I would always be in the process of swallowing drugs. Because pain is permanent, so drugs are useless.*  (Sharon) **

Pain was often perceived as a normal phenomenon even after the end of breast cancer treatment. For some women, pain meant that the healing process was running its course. In that case, pain indicated that the treatment was working well, although some women remained cautious since they believed that pain could also indicate a relapse.

*There was nothing we could do, the pain was so intense… Because the cells in my spinal cord are reactivating… So I've got pain in my back, in my pelvis, in places where the cells are being renewed…*  [Do you feel that pain was a good sign?]  *Yes it was proof that everything was reactivated, it was being renewed.*  (Stella) **

In some cases, the normalization of pain was also fuelled by talks with physicians:

**[The last time you saw the doctors, did you tell them that you feel pain?]  *Oh yes, yes, they told me it was normal. They said this was normal. They told me: this is not a small operation you had.*  [Do you think it's normal to feel pain in your case?]  *If I am told that it's normal, I believe what I'm told.*  (Eva) **

**[Does your breast hurt you?]  *Yes it hurts me. It hurts me a lot.*  [And to relieve the pain, what can you do?]  *Nothing.*  [Have you told your doctor?]  *Sure, he said it's normal. In the end I wonder, as I have pain everywhere, maybe breast pain is normal? Given that I feel pain in my hands, because I have osteoarthritis… It must be part of a more generalized pain.*  (Linda) **

Some participants reported having told their physicians that they were suffering pain and they were sent to a psychiatrist because pain was viewed as a kind of depressive symptoms.

**[Did you consult a physician for pain?]  *Oh yes, I saw an algo…*  [An algologist?]  *Yes. Very nice indeed. They sent me to a psychiatrist because they said that it was all in my head, that it was my mind that wasn't working. But for six months I was in terrible, terrible, terrible pain. Here and here, look. So I quickly phoned my doctor. He said “no”, you know, “you should not have pain there”. And even now, occasionally, I feel such pain.*  (Eva) **

Such “psychiatrization” of pain made women feel impotent and guilty because it implied that their pain was not “real”. Moreover, some participants clearly lacked information about pain. For example, one of them suffered from phantom pain but was never told about such a condition, or at least did not remember being told about it:

*I had the feeling of having a breast for quite some time, like when one loses a member, I felt pain when I had nothing. And it is psychological, is not it? I felt it was still there and it was hurting me.*  [Yes, it is called “phantom pain”, the doctors did not mention it?]  *No.*  (Nancy) **

### 3.4. Dealing with Pain in Daily Life

Before considering behavioral adaptation to chronic pain, it is important to mention that women's psychological adaptation was based on “relativization”. All participants were prone to compare their medical condition, and their pain in particular, with other patients' medical conditions and pains. Some women compared themselves with other patients who had been severely burned by radiotherapy and suffered more than them. Others compared themselves with a relative or a friend suffering from a more painful cancer. Older women also tended to compare their then current chronic pain with other pains endured during their lives, for example, childbirth pain. Sometimes physicians encouraged such “relativization”, for example, by invoking the case of leukemic children.

*When I saw myself in this state I thought: there are some people who are worse off. So then I told myself: I have no right to complain. Even now it is one of the principles that govern my life. There is always someone worse off than yourself. There are those who do not have the chance to live.*  (Stella) **

*I prefer to be like this rather than in a wheelchair. There are some who are more unfortunate than me. Not thinking only about myself comforts me.*  (Mary) **

*My boyfriend's situation is worse than mine, he had mouth cancer. They ripped out all his teeth and now he has a special apparatus because he cannot eat, he cann't chew, he must suffer a lot.*  (Eva) **

*How was the radiotherapy? Oh it was…, I wasn't burned too much, I had severe rubeosis, I peeled a little but, well… compared to others I feel very happy.*  (Linda) **

*Yes, there are difficult moments. But you see, I had two small pupils who had leukemia. We went to visit them at the hospital with my husband, and then unfortunately, one passed away. She was 9 years old. And seeing all these little children, with these large perfusions… You know, when I start to complain about my pain, I think about her… And I feel I have no right to complain.*  (Linda) **

*There is a doctor who told me “you know, if you feel pain, madam, take a short tour of accident and emergency and you'll see, you will immediately get better”. He said “go and see a few kids at A&E, you'll stop complaining all the time”… I was so shocked that I never returned to that hospital.*  (Nancy) **

*Women get used to suffering when they give birth, all these things, right? I think a woman is more tolerant of pain than a man. This has been proved. Because more than once my husband said to me: what you've been through, I couldn't have ever borne.*  (Nancy) **

But daily adaptation to chronic pain was also very concrete. Many women gave up some domestic or leisure activities because they could no longer perform them. They also learned to take some precautions, or to carry out some actions differently in less painful ways. In all, daily adaptation to chronic pain was seen as a painful, self-taught, and learning-by-doing process.

**[What kind of domestic activities did you stop doing because of pain?]  *Cleaning, ironing, washing the windows of course, and I cann't drive on long trips. I have to put a ball on the steering wheel so I don't force my muscles.*  (Nancy) **

*When I do a little unusual movement that doesn't go in the direction of the muscle, I get blocked, it's a pain that paralyzes me. So sometimes I'm stuck, and I must wait until the muscle relaxes and then I feel better.*  (Cindy) **

*With my arms I cannot do anything, I can do absolutely nothing. I always have a small paper in my purse that says that if I have an accident, nothing should be done to this arm.*  (Eva) **

*You know, I learned to change some of my movements. I learned movements that relieve. Instead of wringing the kitchen glove like that, now I wring it like this, against the side of the sink.*  (Linda) **

*Trying to avoid feeling pain is really important in every day movements. In my case it is primarily the hands, it's really a problem. At first I tried to forget the pain, but it quickly brought me back to reality. So pain forced me to think. Especially about some very ordinary movements. For example, now, I've got used to holding my cup with both hands.*  (Linda) **

## 4. Discussion

Before discussing our results, some limitations of the present study must be acknowledged. First, we only interviewed a small sample of women, in two specific age ranges, 24 months after diagnosis, and within a region-based cohort of breast cancer survivors. As a consequence, our results, and especially the prevalence of posttreatment chronic pain, should not be generalized to the wider population, or to other cancers. Secondly, we only interviewed patients, not their physicians: pain narratives should be compared with what physicians' have to say, especially concerning the therapeutic relationship. Third, the aim of the study was not to call the validity of the WHOQOL-BREF questionnaire into question. This psychometric tool is not designed to assess cancer pain, unlike other cancer-specific questionnaires. Our aim was rather to illustrate the relative invisibility of chronic pain when a routinely QoL assessment tool is used.

Iatrogenic pain endured by cancer survivors used to be viewed as a relatively unimportant and unavoidable side-effect of necessary life-saving treatments [21]. Even though this perception is changing, our results were in line with previous findings that pointed out that such pain is still frequently underestimated since it remains frequently hidden by patients and is neglected by healthcare professionals [22–24]. Of course, it is difficult to share one's pain experience, especially using a closed-ended questionnaire: respondents answering questions in pain surveys frequently write unsolicited comments on the margins of questionnaires [25].

Previous studies showed that cancer patients tend to believe that pain is inevitable [24, 26], and to equate chemotherapy toxicity with its efficacy [27, 28]. Our results broaden these conclusions to other treatments side-effects, and to patients who had undergone surgery and had completed chemotherapy and/or radiotherapy. For example, a previous qualitative study conducted in the early 1990s in the Netherlands among surgical breast cancer patients found that many of them concealed their postoperative pain and did not ask for pain medication [29]. Such inhibition in reporting pain was due to several factors related to both patients and nurses: many patients believed that postoperative pain was inevitable, they did not want to become used to taking painkillers, and nurses fuelled their belief by suggesting that such pain was normal and did not require alleviation. Nearly two decades later, we find similar results in France. But our results suggest that physicians, and not only nurses, are also involved in the “normalization” of pain. More importantly, we found that this process of pain “normalization” and the resulting under-treatment of pain continue long after hospitalization.

In addition, this process of pain normalization appeared to be fuelled by very contrasting ways in which patients give meaning to their pain: pain could be considered as a transient condition, a necessary step toward recovery, a proof that the treatment is effective, or on the contrary it can be viewed as a permanent condition people have to get accustomed to. Moreover, our results suggest that physicians did not provide enough information concerning pain, and sometimes might even fuel inadequate pain-related beliefs, instead of fighting them. This is of great significance especially in view of the importance of the doctor-patient relationship in shaping patient beliefs toward pain and pain management [26].

## 5. Conclusions

During in-depth interviews, half the participants reported significant chronic pain remaining 24 months after breast cancer diagnosis. Most of the time, this pain was not captured by the WHOQOL questionnaire, and it continued without medical care. Pain was “normalized” in various ways which contributed to preventing such care being given: it was considered either as a necessary step on the road to recovery, as a permanent condition one must learn to live with, as the sign that completed medical treatments are working or as a depressive symptom. All participants had the tendency to put their pain into perspective, comparing themselves with other patients suffering worse pain. They also learned to deal with pain in their daily lives, by taking precautions, giving up some activities, and changing the way they performed certain normal physical movements.

Breast cancer survivors should be better informed about chronic pain and the ways to alleviate it. Physicians should be involved in this process, and they should also contribute to fighting pain-related beliefs which lead some patients to hide their pain. Finally, apart from pharmacological pain management, techniques to cope with chronic pain in daily lives should be promoted. 

## Figures and Tables

**Table 1:** Sociodemographic and medical characteristics of the 21 breast cancer survivors interviewed.

	Age	PHY	PSY	Marital status	Employment situation	Mastectomy	Reconstruction	Axillary surgery	On hormonotherapy at the time of the study
Stella	26	28	15	Single	Working	Partial	No	Yes	Yes
Cindy	26	24	16	Single	Working	Partial	No	No	Yes
Hannah	30	29	23	Married	Working	Partial	No	No	Yes
Emmy	31	31	24	Married	Sick leave	Partial	No	Yes	No
Megan	32	32	20	Married	Working	Partial	No	No	Yes
Helen	32	30	25	Married	Working	Partial	No	No	No
Nancy	33	21	8	Married	Working	Partial	Yes	Yes	Yes
Sue	40	34	29	Married	Working	Total	Yes	Yes	Yes
Silvia	41	27	22	Married	Working	Total	Yes	Yes	Yes
Kate	42	26	23	Married	Housewife	Total	Yes	Yes	Yes
Hillary	42	35	29	Married	Working	Partial	Yes	Yes	Yes
Laura	42	23	20	Married	Working	Total	Yes	Yes	Yes
Rachel	43	15	12	Single	Sick leave	Partial	No	Yes	No

Bree	66	29	23	Widow	Retired	Partial	No	No	Yes
Eva	68	21	11	Widow	Retired	Partial	Yes	No	Yes
Linda	68	26	23	Married	Retired	Partial	No	Yes	Yes
Mary	72	35	27	Married	Retired	Partial	No	No	Yes
Clara	75	15	17	Divorced	Retired	Total	No	Yes	Yes
Ella	75	26	23	Married	Retired	Partial	No	No	Yes
Sharon	78	24	20	Single	Retired	Partial	No	Yes	Yes
Susan	83	27	22	Married	Retired	Total	No	Yes	Yes

**Table 2:** WHOQOL-BREF items, pain management, and pain narratives.

	QoL self-rating	Feeling activity limitation due to pain	Satisfaction in everyday activities	Pain management	Pain-related quotations from in-depth interviews
Clara	Neither…^1^	Very much	Neither…^2^	Paracetamol	My scar hurts … I have also shooting pains in the arm, I can't lift it.
Ella	Good	A little	Neither…^2^	Diclofenac	I'm dripping with sweat … I've lost my appetite … and the pain starts every morning at 7 A.M.
Nancy	Very poor	A little	Neither…^2^	None	My scar hurts all the time, especially when the weather is hot … The scar was done too tight and it hurts a lot because I put on weight due to my pills.
Eva	Poor	Not at all	Satisfied	Diclofenac, paracetamol	I have a hematoma since breast reconstruction … it has been hurting for six months now … I'm tired as soon as I wake up and I feel aching in my entire body, especially the bones.
Linda	Good	Not at all	Neither…^2^	Trinitrin	My breastbone hurts a lot … sometimes my chest feels like it has a huge weight on it and I suffocate … I can't sleep.
Emmy	Very good	A little	Satisfied	None	I'm always out of breath because of the chemotherapy, it's weakened my heart … I'm 31 and I can't ride a scooter, I can't raise my arms … I can't lift a pack of milk, it's too painful.
Stella	Good	Moderate	Satisfied	None	My arm has been hurting since the surgery … especially in the morning … and sometimes my breast also hurts.
Cindy	Poor	Moderate	Satisfied	None	Before my cancer I used to blow dry 20 clients' hair every day, but now after 4 or 5 I must stop because my arm hurts too much … today I can't even hold my handbag … sometimes I've got shooting pains in my breast, like a stab from a knife.
Rachel	Neither…^1^	Very much	Not satisfied at all	Paracetamol	I can't go upstairs, my ankles swell … If I lift something my arm starts hurting and swelling the day after.
Silvia	Good	Moderate	Satisfied	None	Because of the pills I take I have muscular pain, like cramps, especially in the back and the arm.

1: Neither poor nor good.

2: Neither satisfied nor dissatisfied.

## References

[1] McPherson T (2003). Impact on the quality of life of lymphoedema patients following introduction of a hygiene and skin care regimen in a Guyanese community endemic for lymphatic filariasis: a preliminary clinical intervention study. *Filaria Journal*.

[2] Trétarre B, Guizard AV, Fontaine D (2004). Cancer du sein chez la femme: incidence et mortalité, France 2000. *Bulletin Epidémiologique Hebdomadaire*.

[3] Coleman MP, Gatta G, Verdecchia A, Estève J, Sant M (2003). EUROCARE-3 summary: cancer survival in Europe at the end of the 20th century. *Annals of Oncology*.

[4] Brenner H, Francisci S, de Angelis R (2009). Long-term survival expectations of cancer patients in Europe in 2000–2002. *European Journal of Cancer*.

[5] Alfano CM, McGregor BA, Kuniyuki A (2006). Psychometric evaluation of the brief cancer impact assessment among breast cancer survivors. *Oncology*.

[6] Pearce NJM, Sanson-Fisher R, Campbell HS (2008). Measuring quality of life in cancer survivors: a methodological review of existing scales. *Psycho-Oncology*.

[7] Le Coroller-Soriano AG, Malavolti L, Mermilliod C (2008). *La vie deux ans après le Diagnostic de Cancer*.

[8] Den Oudsten BL, Van Heck GL, Van der Steeg AFW, Roukema JA, De Vries J (2009). Predictors of depressive symptoms 12 months after surgical treatment of early-stage breast cancer. *Psycho-Oncology*.

[9] Lee VC, Rowlingson JC, Spilker B (1990). Chronic pain management. *Quality of Life Assessments in Clinical Trials*.

[10] Vissers KCP, Besse K, Wagemans M (2011). Pain in patients with cancer. *Pain Practice*.

[11] Caffo O, Amichetti M, Ferro A, Lucenti A, Valduga F, Galligioni E (2003). Pain and quality of life after surgery for breast cancer. *Breast Cancer Research and Treatment*.

[12] Burckhardt CS, Jones KD (2005). Effects of chronic widespread pain on the health status and quality of life of women after breast cancer surgery. *Health and Quality of Life Outcomes*.

[13] Diel IJ (2007). Effectiveness of bisphosphonates on bone pain and quality of life in breast cancer patients with metastatic bone disease: a review. *Supportive Care in Cancer*.

[14] Daut RL, Cleeland CS (1982). The prevalence and severity of pain in cancer. *Cancer*.

[15] Greenwald HP, Bonica JJ, Bergner M (1987). The prevalence of pain in four cancers. *Cancer*.

[16] Marchettini P (2008). More on pain semantics. *European Journal of Pain*.

[17] World Health Organization WHOQoL Study Protocol.

[18] World Health Organization *WHOQoL-BREF, Introduction, Administration, Scoring and Generic Version of the Assessment*.

[19] Glaser B, Strauss A (1967). *The Discovery of Grounded Theory*.

[20] Charmaz K (2006). *Constructing Grounded Theory: A Practical Guide Through Qualitative Analysis*.

[21] Morgan PA, Franks PJ, Moffatt CJ (2005). Health-related quality of life with lymphoedema: a review of the literature. *International Wound Journal*.

[22] Hare M (2000). The lived experience of breast cancer-related lymphoedema. *Nursing Standard*.

[23] Arman M, Rehnsfeldt A, Lindholm L, Hamrin E (2002). The face of suffering among women with breast cancer—being in a field of forces. *Cancer Nursing*.

[24] Arman M, Rehnsfeldt A (2003). The hidden suffering among breast cancer patients: a qualitative metasynthesis. *Qualitative Health Research*.

[25] Smith MV (2008). Pain experience and the imagined researcher. *Sociology of Health and Illness*.

[26] Dawson R, Spross JA, Jablonski ES (2002). Probing the paradox of patients’ satisfaction with inadequate pain management. *Journal of Pain and Symptom Management*.

[27] Rosman S (2004). Cancer and stigma: experience of patients with chemotherapy-induced alopecia. *Patient Education and Counseling*.

[28] Bell K (2009). ‘If it almost kills you that means it’s working!’ cultural models of chemotherapy expressed in a cancer support group. *Social Science and Medicine*.

[29] Francke AL, Theeuwen I (1994). Inhibition in expressing pain: a qualitative study among Dutch surgical breast cancer patients. *Cancer Nursing*.

